# Ventral Anterior–Lateral Complex of the Thalamus Mediates Chronic Stress‐Induced Pain Hypersensitivity and Underlies Electroacupuncture Analgesia

**DOI:** 10.1002/brb3.70855

**Published:** 2025-09-09

**Authors:** Jie Chen, Qinling Li, Wei Yu, Min He, Zui Shen, Peipei Feng

**Affiliations:** ^1^ Tongde Hospital of Zhejiang Province Affiliated to Zhejiang Chinese Medical University（Tongde Hospital of Zhejiang Province） Hangzhou China; ^2^ Key Laboratory of Acupuncture and Neurology of Zhejiang Province, Department of Neurobiology and Acupuncture Research The Third Affiliated Hospital of Zhejiang Chinese Medical University Hangzhou China

**Keywords:** analgesia, chronic restraint stress, electroacupuncture, pain, ventral anterior–lateral complex of the thalamus

## Abstract

**Background:**

Mental disorders frequently co‐occur with pain, yet pain mechanisms in non‐peripheral etiologies (e.g., chronic psychological stress) remain underexplored. The ventral anterior–lateral thalamic complex (VAL) is implicated in emotional processing, but its role in chronic stress‐induced pain hypersensitivity is unclear. Electroacupuncture (EA) is clinically used for pain management, but its efficacy and mechanisms in chronic stress‐driven pain hypersensitivity require validation.

**Methods:**

A chronic restraint stress (CRS) model was established in male mice. Behavioral assessments were performed to quantify mechanical sensitivity (hindpaws and abdomen using von Frey filaments), thermal sensitivity (hot plate test), and spontaneous pain‐like behaviors. Bidirectional chemogenetic approaches targeted VAL CaMKIIα‐positive neurons. EA was applied at Zusanli (ST36) and Sanyinjiao (SP6) acupoints.

**Results:**

CRS stably induced pain hypersensitivity phenotypes, including mechanical allodynia (hindpaws/abdomen), thermal hyperalgesia, and spontaneous pain‐like behaviors. Chemogenetic inhibition of VAL CaMKIIα‐positive neurons reversed these CRS‐induced hypersensitivity responses. Conversely, activating these neurons in naive mice recapitulated the full spectrum of hyperalgesia phenotypes. EA alleviated CRS‐induced hindpaw mechanical/thermal hyperalgesia, abdominal allodynia, and spontaneous pain. EA's effects on hindpaw mechanical/thermal hyperalgesia were mediated by suppression of VAL CaMKIIα‐positive neurons. In contrast, its amelioration of abdominal allodynia and spontaneous pain persisted despite chemogenetic activation of VAL CaMKIIα‐positive neurons, indicating possible distinct pathways.

**Conclusion:**

This study reveals the pivotal role of thalamic VAL CaMKIIα‐positive neurons in chronic stress‐associated pain hypersensitivity and elucidates EA's analgesic mechanisms, providing novel therapeutic strategies for emotion–pain comorbidity.

## Graphical abstract

Chronic restraint stress (CRS) necessarily and sufficiently induces hyperalgesia by activating ventral anterior–lateral complex of the thalamus (VAL) CaMKIIα‐positive neurons. Electroacupuncture alleviates CRS‐induced paw mechanical allodynia and thermal hyperalgesia by suppressing these VAL neurons.

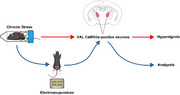

## 1 Introduction

Mental disorders are frequently comorbid with clinically significant somatic manifestations, particularly pain (Henning et al. 2020; Penninx et al. 2021; Szuhany and Simon 2022). Patients with current or remitted mental disorders exhibit substantially higher prevalence of chronic pain compared to non‐affected controls (de Heer et al. 2014). Emerging evidence suggests that emotional states function as potent neuromodulatory stressors within the pain matrix (Ahmad and Zakaria 2015). Negative emotions, such as preoperative anxiety, not only trigger or exacerbate pain but also predispose patients to heightened postoperative pain experiences (Wu et al. 2019). In contrast, positive emotions relieve pain, and suppressing negative emotions may be the optimal way to prevent pain (Müller et al. 2022). These clinical observations underscore the imperative to investigate pain mechanisms in mental disorders; otherwise, unresolved pain could reciprocally exacerbate the underlying psychological conditions, creating a self‐reinforcing pathological loop.

The ventral anterior–lateral complex of the thalamus (VAL) is a subregion mediating emotional and motivational drive (Haber and Calzavara 2009). Our previous study revealed that VAL is critically involved in anxiety‐related processing (He et al. 2024; Shen et al. 2021). Clinical studies demonstrate that anxiety symptoms induced by painful experiences show strong correlations with VAL dysfunction (Casteen et al. 2022), and deep brain stimulation targeting VAL significantly alleviates anxiety in patients (Huys et al. 2016). Furthermore, human neuroimaging studies indicate that hyperactivity in the VAL is associated with enhanced emotion‐modulated pain perception (Wagner et al. 2009). However, the precise mechanisms through which VAL neuronal activity mediates chronic stress‐driven pain hypersensitivity remain to be elucidated.

Electroacupuncture (EA) is a widely accepted non‐pharmacological intervention for clinical pain management (Candon et al. 2024; W. X. Li et al. 2025). Previous preclinical investigations of EA analgesia have primarily focused on peripherally injury‐driven pain models, including inflammatory and neuropathic pain induced by complete Freund's adjuvant (Wan et al. 2025) or nerve ligation (Chen et al. 2025). However, whether EA exerts analgesic efficacy specifically on mental disorder‐associated hyperalgesia—particularly emotion‐driven pain without peripheral injury—and the neural mechanisms underlying such effects require rigorous preclinical validation. Clarifying these questions is critical to inform future clinical trials targeting chronic stress‐induced pain syndromes.

In this study, we first employed a chronic restraint stress (CRS) model to induce multifaceted hyperalgesic phenotypes in mice, including bilateral hindpaws mechanical allodynia, thermal hyperalgesia, abdominal mechanical allodynia, and spontaneous pain‐like behaviors, followed by comprehensive behavioral assessments of these sensory abnormalities. Building on this, we utilized chemogenetic approaches to specifically inhibit VAL CaMKIIα‐positive neurons in CRS mice, evaluating their modulatory effects on multidimensional pain hypersensitivity. In parallel, chemogenetic activation of VAL CaMKIIα‐positive neurons in naive mice was performed to determine their sufficiency in inducing hyperalgesic states. Finally, we examined the therapeutic efficacy of EA on CRS‐induced hyperalgesia and whether its analgesic effects are mediated through VAL CaMKIIα‐positive neurons.

## 2 Materials and Methods

### 2.1 Animals

All male C57BL/6J mice (aged 8–12 weeks) were purchased from Vital River Laboratories (Jiaxing, China) and housed four to five per cage. The animals were maintained under standardized conditions with controlled temperature (23°C–25°C), humidity (40%–60%), and a 12‐h light–dark cycle (lights on at 8:00 a.m.), with free access to food and water. All animal care and experimental procedures followed the National Institutes of Health Guidelines for the Care and Use of Laboratory Animals. The experimental protocol was approved by the Laboratory Animal Management and Welfare Ethics Committee of Zhejiang Chinese Medical University (IACUC approval number), and all studies complied with relevant ethical regulations for animal research.

### 2.2 CRS Model

Ventilation holes were drilled in 50‐mL EP tubes to ensure adequate airflow for restrained mice. Experimental mice were subjected to daily 12‐h restraint (8:00 p.m. to 8:00 a.m.) in the modified tubes throughout the study duration. Control cohorts underwent matched food and water deprivation during the restraint period while maintaining unrestricted movement within their home cages.

### 2.3 Hindpaw Mechanical Allodynia Assessment

Mechanical withdrawal thresholds of bilateral hindpaws were quantified using the up–down method with calibrated von Frey filaments (0.02, 0.04, 0.07, 0.16, 0.4, 0.6, 1.0, 1.4, and 2.0 g; Touch‐Test Sensory Evaluator, North Coast Medical, Gilroy, CA). Testing occurred under standardized conditions: ambient temperature 24 ± 1°C, humidity 50 ± 10%, and background noise < 40 dB.

Pretest preparations included thorough cleaning of the elevated wire mesh platform and opaque glass chambers with 75% ethanol/water solution to eliminate olfactory cues. Mice were acclimatized in chambers for 30 min prior to testing. Filaments were applied perpendicular to the plantar surface with sufficient force to bend the fiber for 2 s. Positive responses (×) were recorded for paw withdrawal/flinching/licking behaviors, triggering the use of the next weaker filament. Negative responses (○) prompted progression to stronger filaments. Withdrawal thresholds were calculated using the formula: Mechanical withdrawal threshold (*g*) = 10 ^ (*xf* × *k* × *δ* − 4).

### 2.4 Thermal and Cold Hyperalgesia Assessment (Hot/Cold Plate Test)

Thermal and cold nociceptive responses were evaluated using a temperature‐controlled analgesia meter (Model 35100, UGO BASILE). For thermal testing, the platform was maintained at 52.0 ± 0.5°C, while cold sensitivity assessments were conducted at 4.0 ± 0.5°C. Pretest protocols included cleaning the heated or cooled surface and acrylic enclosure with 75% ethanol to eliminate olfactory interference. Behavioral responses were recorded via an integrated video tracking system.

In the hot plate test, withdrawal latency was defined as the time to first nocifensive behavior (paw withdrawal, licking, or jumping) within a 30‐s cutoff. Mice not responding within 30 s were removed, with latency recorded as 30 s. For the cold plate test, nociceptive latency was measured as the time to first observable pain‐related behavior (paw withdrawal, licking, or struggling) within a 60‐s window, and nonresponsive mice were removed at 60 s (maximal latency).

Both paradigms involved three trials per animal (≥ 10 min intertrial intervals), with mean withdrawal latency calculated from triplicate measurements.

### 2.5 Spontaneous Pain Assessment

Prior to each test, the elevated wire mesh platform and opaque glass chambers were wiped with 75% alcohol and water to prevent odor interference. Mice were placed in chambers for 35 min acclimatization followed by 5 min video recording. Behavioral parameters were analyzed: paw withdrawal latency (time to first withdrawal), total withdrawal duration, and hindpaw licking frequency were quantified.

### 2.6 Abdominal Mechanical Allodynia Assessment

Abdominal cutaneous mechanical thresholds were quantified using the up–down method with von Frey filaments (0.02, 0.04, 0.07, 0.16, 0.4, 0.6, 1.0, 1.4, and 2.0 g). Pretest procedures included shaving abdominal hair to expose the skin surface. Mice were acclimatized in opaque chambers for 30 min prior to testing. Filaments were applied perpendicular to the abdominal skin for 2 s. Positive responses (×) were recorded for abdominal contraction, licking, or struggling behaviors, followed by testing with weaker filaments. Negative responses (○) prompted the use of stronger filaments. Thresholds were calculated using the established up–down algorithm.

### 2.7 Stereotaxic Viral Delivery

Following anesthesia induction, mice were secured in a stereotaxic frame (Model 68513, RWD Life Science, China). After skull leveling, viral solution was infused into the bilaterally VAL (AP: −0.95 mm, ML: ±1.1 mm, DV: −3.6 mm) at 40 nL/min using a 10‐µL syringe (World Precision Instruments) coupled to a microinjection pump (Legato 130, KD Scientific). Postinjection scalp incisions were sutured, and animals were returned to home cages upon regaining consciousness.

To modulate VAL CaMKIIα‐positive neuronal activity, inhibitory (AAV‐CaMKIIα‐hM4Di‐mCherry, 5.71×10^12^ vg/mL) or excitatory (AAV‐CaMKIIα‐hM3Dq‐mCherry, 5.25×10^12^ vg/mL) DREADD vectors were bilaterally injected (60 nL/site; BrainVTA, China).

### 2.8 Chemogenetic Manipulation

To validate the impact of VAL CaMKIIα‐positive neuronal activity modulation on hyperalgesia in CRS or naive mice, clozapine‐N‐oxide (CNO, 2 mg/kg, BrainVTA) was intraperitoneally administered 30 min prior to behavioral assessments.

For assessing EA's potential VAL CaMKIIα‐dependent mechanisms, CNO was delivered 30 min prior to EA intervention. Control cohorts received equivalent 0.9% saline injections following identical protocols.

### 2.9 Immunohistochemistry and Viral Expression Verification

Mouse brain sampling was performed the day after all behavioral studies were done. Mice were anesthetized and transcardially perfused with 0.9% saline followed by 4% (w/v) paraformaldehyde (PFA). Brains were postfixed in 4% PFA at 4°C overnight, then sequentially dehydrated in 15% and 30% sucrose solutions. Coronal sections (30 µm) were cut using a cryostat (CryoStar NX50 HOP, Thermo Fisher Scientific) and mounted with DAPI‐containing fluorescent mounting medium (ab104139, Abcam). Sections were imaged using a ZEISS Axio Imager M2 microscope.

For c‐Fos immunofluorescence, sections were rehydrated in PBS and blocked (1 h, 37°C). After blocking solution removal, slices were incubated with rabbit anti‐c‐Fos antibody (1:1000, #226008, Synaptic Systems) at 4°C for 18 h, followed by Alexa Fluor 488‐conjugated secondary antibody (1:500, ab150061, Abcam; 1 h, 37°C). Sections were coverslipped with DAPI medium for nuclear counterstaining. For validation experiments, HM3D‐mediated activation was assessed in naïve mice, while HM4D‐mediated inhibition was evaluated in CRS model mice.

### 2.10 EA Intervention

Mice received EA treatment on CRS Days 9, 10, and 11. Following restraint fixation, bilateral acupuncture needles (0.16 mm × 7 mm) were inserted at Zusanli (ST36) and Sanyinjiao (SP6) acupoints. The Fang's Transcutaneous‐Acupoint Therapeutic Apparatus (Model FANGS‐100A, Hangzhou Dalishen Medical Device Co. Ltd., China) was connected to needle handles, delivering continuous‐wave stimulation at 100 Hz and 0.3 mA for 30 min.

### 2.11 Anxiety‐Like Behavior Tests

Anxiety‐like behaviors were assessed using the elevated plus‐maze (EPM) and open field (OF) tests. Both tests utilized the ANY‐maze video tracking system (Stoelting, USA) for behavioral recording over 5 min. The apparatus for each test was cleaned with 75% alcohol between subjects to eliminate olfactory cues. All behavioral testing (EPM and OF) was conducted by a dedicated researcher.

### 2.12 Statistical Analysis

**FIGURE 1:**
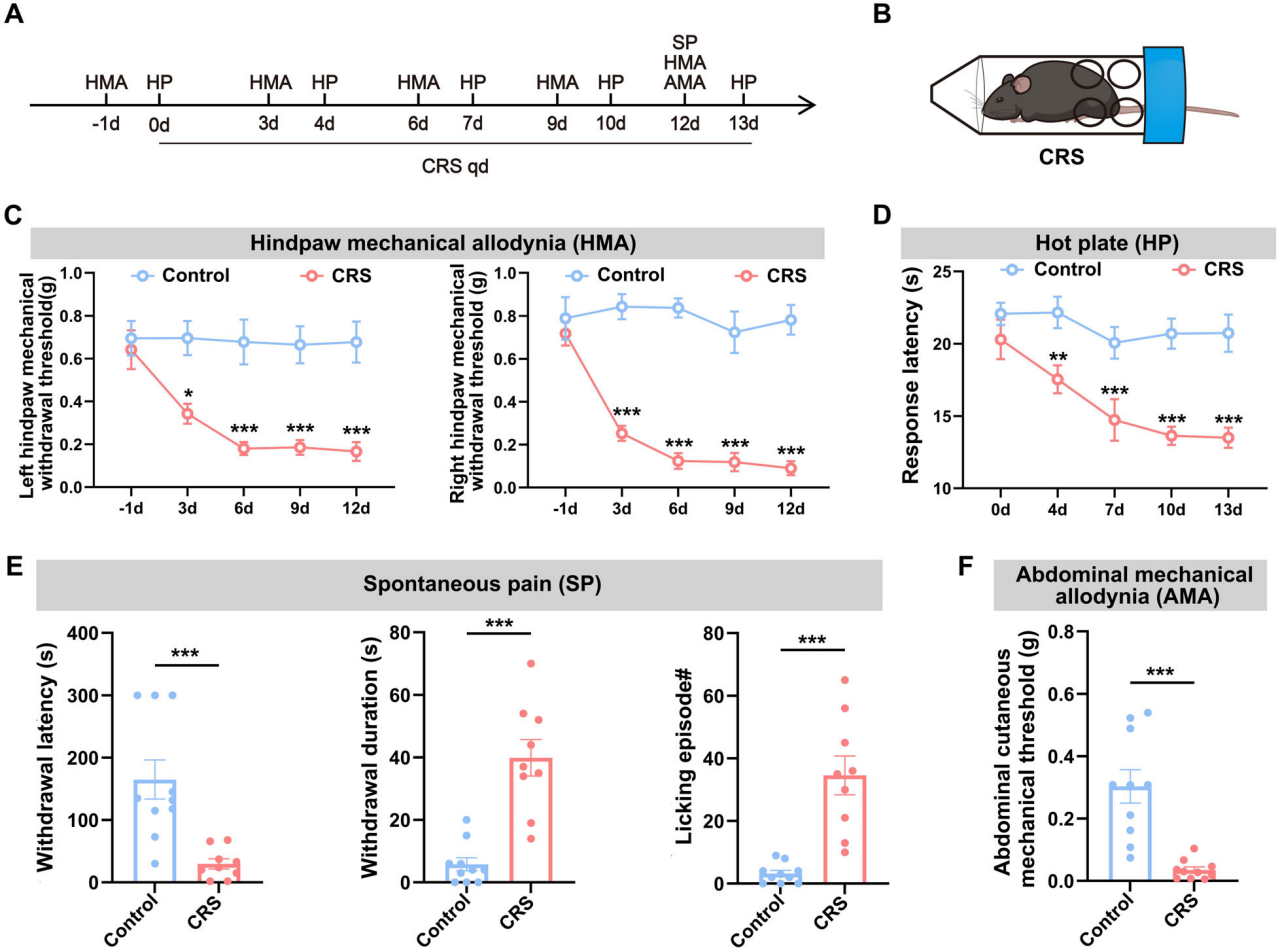
Chronic restraint stress model induces hyperalgesia. (A) Schematic of the experimental design. (B) Schematic diagram of the chronic restraint stress model. (C) Statistical results of 50% paw withdrawal thresholds for the left hindpaw (left) and the right hindpaw (right). (D) Statistical results of hot plate latency. (E) Statistical results of spontaneous pain: paw withdrawal latency (left), withdrawal duration (middle), and number of licking episodes (right). (F) Statistical results of abdominal mechanical pain. All data are expressed as mean ± SEM. In (C) and (D): Two‐way repeated‐measures ANOVA followed by Tukey's post hoc test, compared with the control group at the same time point: *p < 0.05, **p < 0.01, ***p < 0.001. In (E) and (F): Independent samples *t*‐test, ***p < 0.001.

All data are expressed as mean ± SEM. The hindpaw mechanical allodynia (Figure 1C), thermal latency (Figure 1D), and cold sensitivity (Figure S1B) were analyzed using two‐way repeated‐measures ANOVA followed by Tukey's post hoc test. Intergroup comparisons employed Student's *t*‐test. For multigroup analyses, one‐way ANOVA with Tukey's post hoc testing was applied. Statistical significance was set at *p* < 0.05.

## 3 Results

### 3.1 CRS‐Induced Hyperalgesia

To investigate whether CRS induces hyperalgesia in male mice, we systematically assessed nociceptive behaviors across multiple modalities and time points (Figure 1A; Figure S1A). Mechanical allodynia in bilateral hindpaws evaluated using von Frey filaments showed progressive hypersensitivity from Day 3 through Day 12 of CRS (Figure 1C). Thermal hyperalgesia monitored via hot plate tests revealed significantly shortened withdrawal latencies beginning at Day 4 and persisting through Day 13 (Figure 1D), whereas cold plate responses showed no temporal changes (Figure S1B). By Day 12 of CRS intervention, mice developed spontaneous pain‐like behaviors manifested as reduced paw withdrawal latency, prolonged withdrawal duration, and elevated paw‐licking frequency (Figure 1E), concurrent with the emergence of abdominal mechanical allodynia evidenced by decreased abdominal cutaneous mechanical thresholds (Figure 1F). These multimodal assessments demonstrate CRS‐induced hyperalgesia spanning mechanical, thermal, and spontaneous pain domains across distinct developmental timelines. Furthermore, we assessed the behavior of mice using the EPM and OF tests on Days 12 and 13 of the CRS model. Our findings indicate that mice subjected to CRS exhibited pronounced anxiety‐like behaviors (Figure S2).

### 3.2 Chemogenetic Inhibition of VAL CaMKIIα‐Positive Neurons Reverses CRS‐Induced Hyperalgesia

**FIGURE 2:**
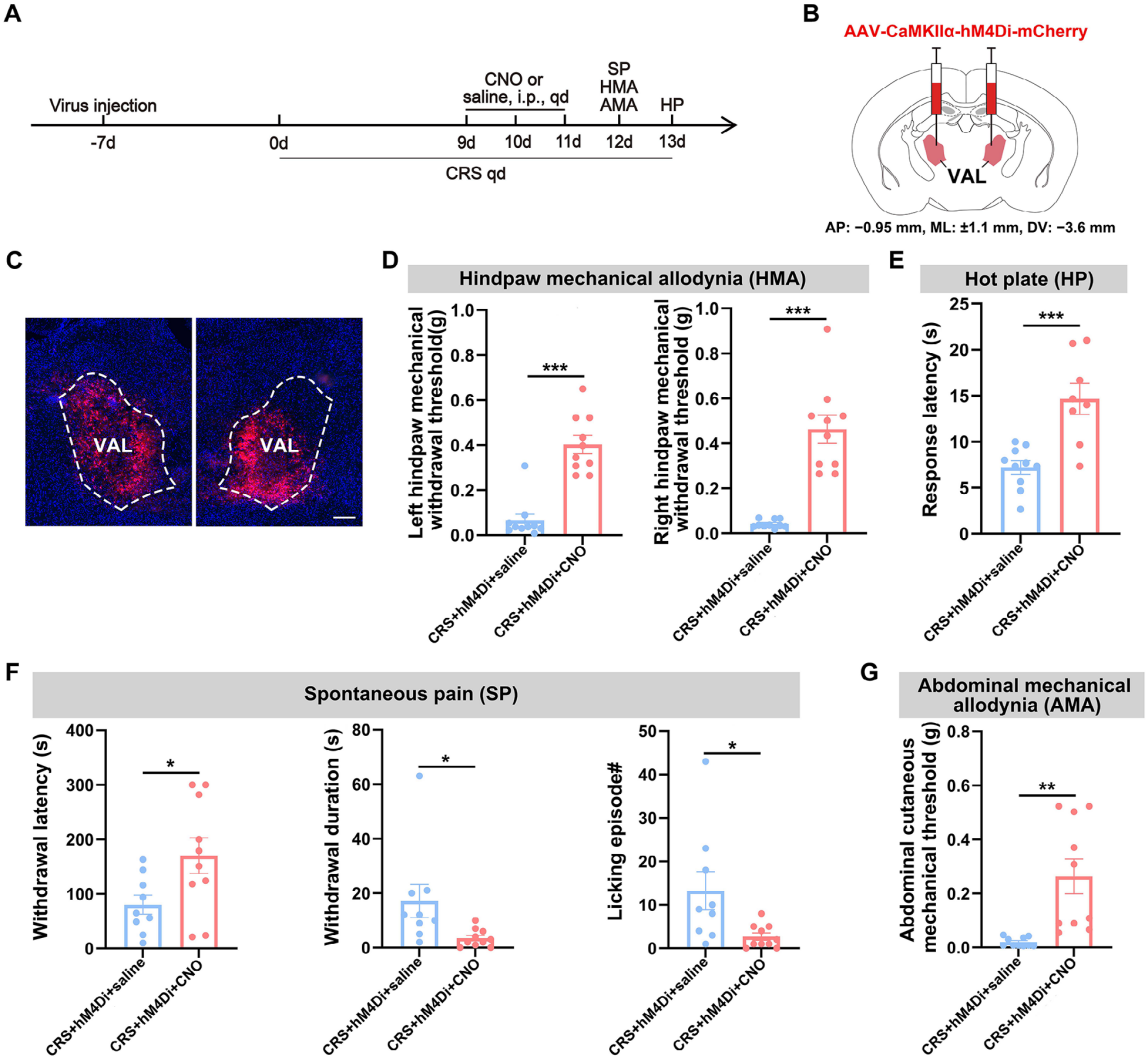
Inhibition of CaMKIIα‐positive neurons in the VAL alleviates hyperalgesia in CRS mice. (A) Schematic of the experimental design. (B, C) Schematic of specific infection of VAL CaMKIIα‐positive neurons with hM4Di‐mCherry. Scale bar: 100 µm. (D) Statistical results of 50% paw withdrawal thresholds for the left hindpaw (left) and the right hindpaw (right). (E) Statistical results of hot plate latency. (F) Statistical results of spontaneous pain: paw withdrawal latency (left), withdrawal duration (middle), and number of licking episodes (right). (G) Statistical results of abdominal mechanical pain. Independent samples *t*‐test for (D–G). All data are expressed as mean ± SEM. *p < 0.05, **p < 0.01, ***p < 0.001.

To determine whether chemogenetic inhibition of CaMKIIα‐positive neurons in the VAL modulates CRS‐induced hyperalgesia, we bilaterally delivered the Gi‐coupled DREADD construct AAV‐CaMKIIα‐hM4Di‐mCherry into the VAL of mice 7 days before CRS initiation, allowing viral expression prior to multimodal behavioral assessments (Figure 2A–C). CRS mice were intraperitoneally administered CNO or saline on Days 9–11 of the stress protocol to suppress neuronal activity (Figure S3A,B). Behavioral assessments performed on CRS Day 12 quantified spontaneous pain‐like behaviors, hindpaw mechanical allodynia, and abdominal mechanical allodynia, with thermal hyperalgesia evaluated on Day 13 (Figure 2A). Compared to control CRS mice, neuronal inhibition significantly elevated mechanical pain thresholds in bilateral hindpaws (Figure 2D) and abdominal regions (Figure 2G), prolonged hot plate withdrawal latencies (Figure 2E), and ameliorated spontaneous pain manifestations through increased paw withdrawal latency, reduced withdrawal duration, and decreased paw‐licking frequency (Figure 2F). These findings collectively demonstrate that suppression of VAL CaMKIIα‐positive neuronal activity alleviates multidimensional hyperalgesia in CRS mice.

### 3.3 Chemogenetic Activation of VAL CaMKIIα‐Positive Neurons Elicits Multidimensional Hyperalgesia in Naive Mice

**FIGURE 3:**
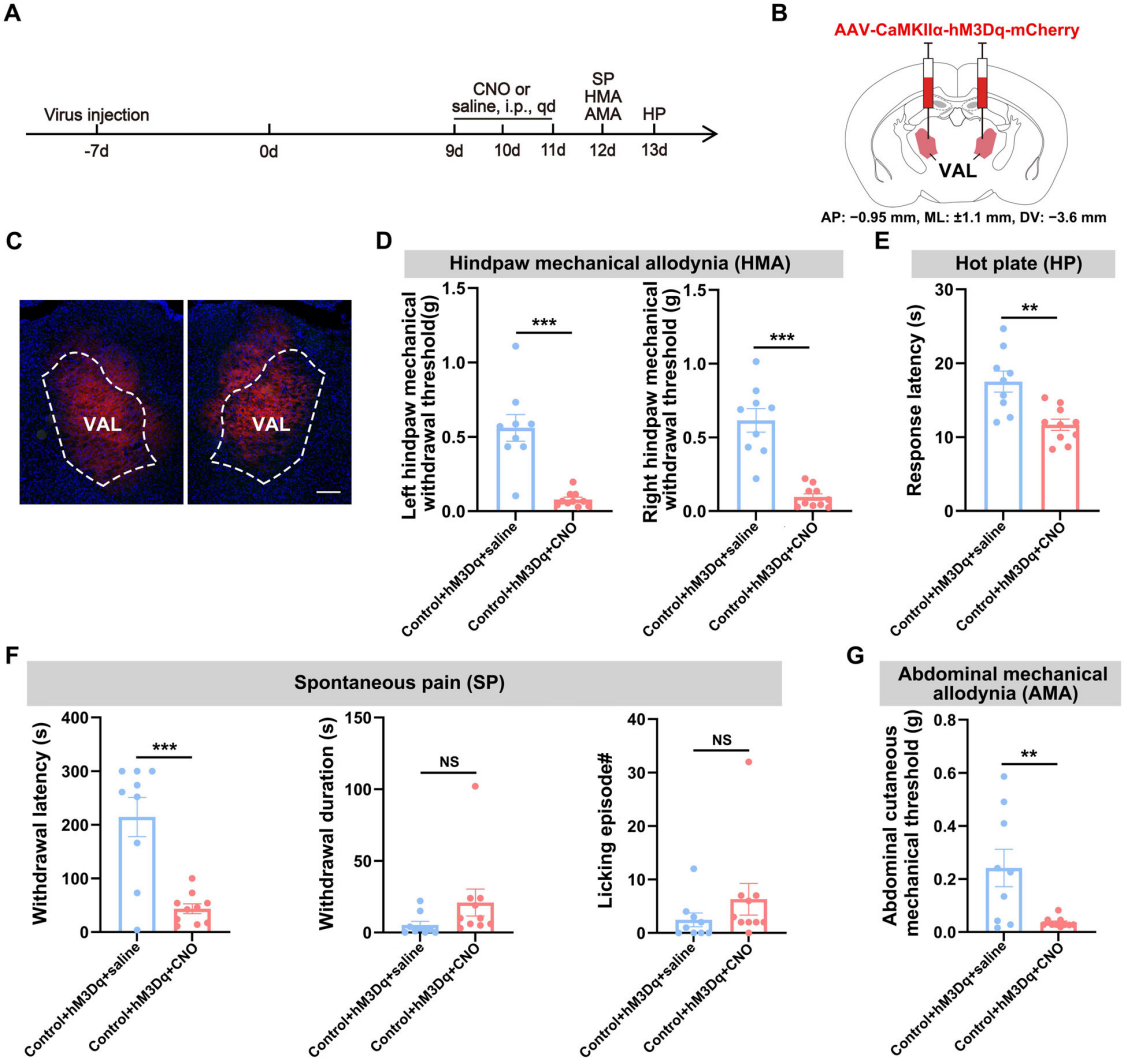
Activation of CaMKIIα‐positive neurons in the VAL induces hyperalgesia in naive mice. (A) Schematic of the experimental design. (B, C) Schematic of specific infection of VAL CaMKIIα‐positive neurons with hM3Dq‐mCherry. Scale bar: 100 µm. (D) Statistical results of 50% paw withdrawal thresholds for the left hindpaw (left) and the right hindpaw (right). (E) Statistical results of hot plate latency. (F) Statistical results of spontaneous pain: paw withdrawal latency (left), withdrawal duration (middle), and number of licking episodes (right). (G) Statistical results of abdominal mechanical pain. Independent samples *t*‐test for (D–G). All data are expressed as mean ± SEM. NS = not significant. *p < 0.05, **p < 0.01, ***p < 0.001.

To determine whether chemogenetic activation of CaMKIIα‐positive neurons in the VAL induces hyperalgesia, we bilaterally delivered viral vectors expressing the Gq‐coupled DREADD hM3Dq (AAV‐CaMKIIα‐hM3Dq‐mCherry) into the VAL of naive mice 19 days before behavioral assessments to achieve cell‐type‐specific activation (Figure 3A–C). Intraperitoneal CNO or saline administration on Days 9–11 activated these neurons (Figure S3C,D), followed by multimodal pain assessments on Days 12 (spontaneous pain, mechanical allodynia) and 13 (thermal sensitivity) (Figure 3A). Neuronal activation significantly reduced mechanical pain thresholds in bilateral hindpaws (Figure 3D) and abdominal regions (Figure 3G), shortened hot plate withdrawal latencies (Figure 3E), and decreased paw withdrawal latency, though withdrawal duration and paw‐licking frequency remained unchanged (Figure 3F). These data demonstrate that selective activation of VAL CaMKIIα‐positive neurons is sufficient to recapitulate key components of hyperalgesia in naive mice.

### 3.4 EA Alleviates CRS‐Induced Hindpaw Mechanical and Thermal Hyperalgesia via Suppression of VAL CaMKIIα‐Positive Neurons

**FIGURE 4:**
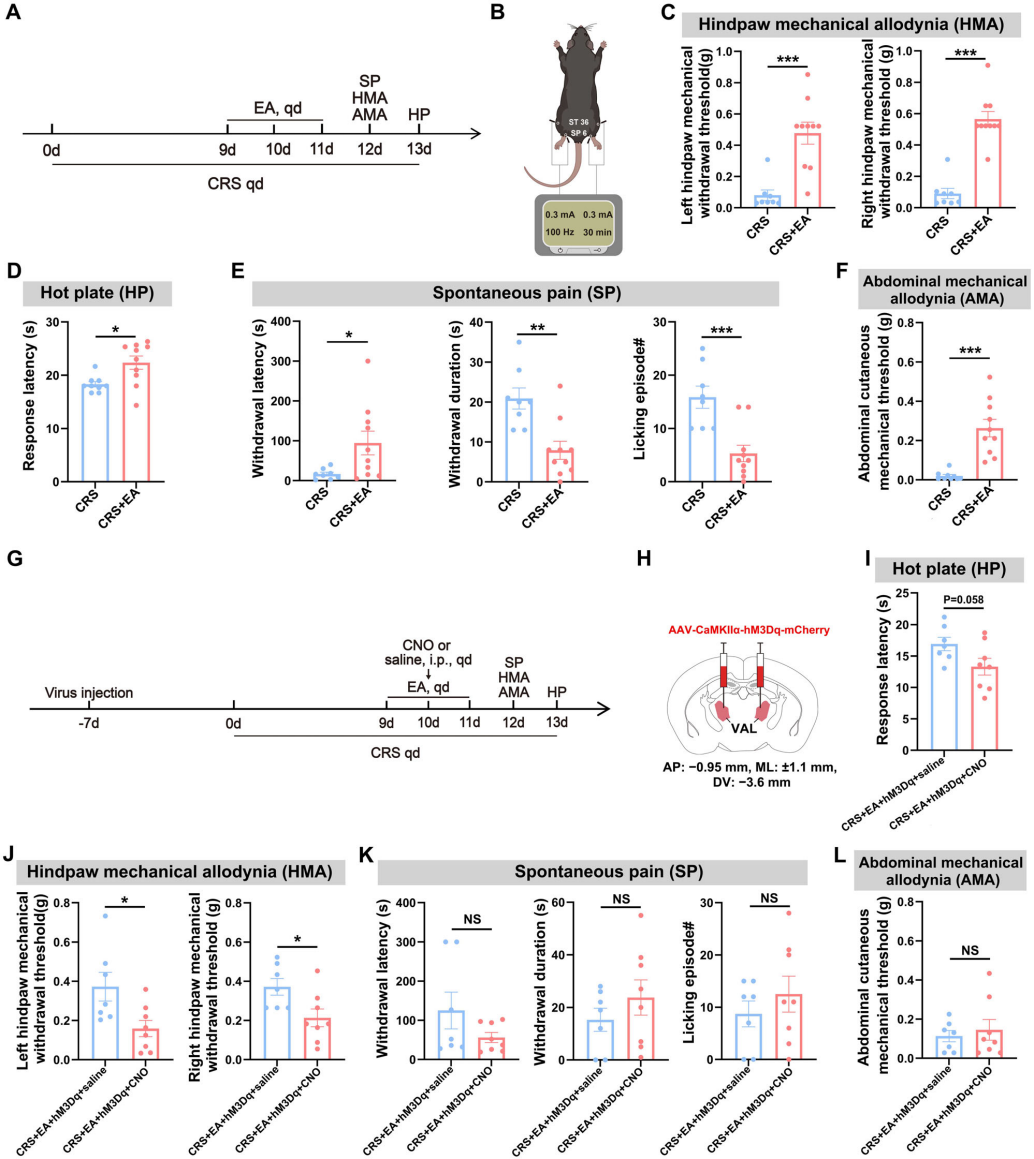
VAL CaMKIIα‐positive neurons are partially involved in EA‐mediated alleviation of hyperalgesia in CRS mice. (A) Schematic of the experimental design. (B) Schematic of EA intervention. (C) Statistical results of 50% paw withdrawal thresholds for the left hindpaw (left) and the right hindpaw (right). (D) Statistical results of hot plate latency. (E) Statistical results of spontaneous pain: paw withdrawal latency (left), withdrawal duration (middle), and number of licking episodes (right). (F) Statistical results of abdominal mechanical pain. (G) Schematic of the experimental design. (H) Schematic of specific infection of VAL CaMKIIα‐positive neurons with hM3Dq‐mCherry. Scale bar: 100 µm. (I) Statistical results of hot plate latency. (J) Statistical results of 50% paw withdrawal thresholds for the left hindpaw (left) and the right hindpaw (right). (K) Statistical results of spontaneous pain: paw withdrawal latency (left), withdrawal duration (middle), and number of licking episodes (right). (L) Statistical results of abdominal mechanical pain. Independent samples *t*‐test for (C–F, I–L). All data are expressed as mean ± SEM. NS = not significant. *p < 0.05, **p < 0.01, ***p < 0.001.

To evaluate the therapeutic effects of EA on CRS‐induced hyperalgesia, mice received bilateral EA stimulation at Zusanli (ST36) and Sanyinjiao (SP6) acupoints (100 Hz, 0.3 mA, 30 min/day) on CRS Days 9–11 (Figure 4A,B). Behavioral assessments conducted on CRS Day 12 revealed significant alleviation of mechanical allodynia in bilateral hindpaws (Figure 4C) and abdominal regions (Figure 4F), while thermal hyperalgesia measured on Day 13 showed prolonged withdrawal latencies (Figure 4D). Spontaneous pain‐like behaviors were also attenuated, evidenced by increased paw withdrawal latency alongside reduced withdrawal duration and licking frequency (Figure 4E). These results confirm EA's capacity to counteract multidimensional hyperalgesia specifically induced by chronic stress.

To investigate the neural substrates underlying EA's suppression of CRS‐induced hyperalgesia, we bilaterally delivered the excitatory chemogenetic vector AAV‐CaMKIIα‐hM3Dq‐mCherry into the VAL (Figure 4G,H). Mice received intraperitoneal CNO injections 30 min prior to EA intervention on CRS Days 9–11, allowing neuronal activation during EA intervention period. Behavioral analyses revealed that chemogenetic activation of VAL CaMKIIα‐positive neurons selectively reversed EA's analgesic effects on hindpaw mechanical thresholds (Figure 4J) and diminished thermal analgesia (Figure 4I), whereas abdominal mechanical sensitivity and spontaneous pain parameters (withdrawal latency, duration, and licking frequency) remained unaffected (Figure 4K,L). These findings demonstrate that EA's alleviation of CRS‐induced hindpaw mechanical/thermal hyperalgesia specifically requires suppression of VAL CaMKIIα‐positive neurons, while its effects on spontaneous pain and abdominal mechanical allodynia are mediated by distinct neural pathways.

## 4 Discussion

In this study, we established a CRS paradigm that robustly induced multifaceted hyperalgesic phenotypes in male mice, including bilateral hindpaw mechanical allodynia, thermal hyperalgesia, abdominal mechanical allodynia, and spontaneous pain‐like behaviors. By employing bidirectional chemogenetic manipulation, we identified VAL CaMKIIα‐positive neurons as pivotal regulators of stress‐induced pain hypersensitivity: Selective inhibition of these neurons in CRS mice reversed multidimensional hyperalgesia, whereas their activation in naive animals recapitulated key pathological pain states, confirming both necessity and sufficiency of this neuronal population in mediating chronic stress‐associated nociceptive sensitization. Notably, EA intervention specifically alleviated CRS‐evoked hindpaw mechanical allodynia and thermal hyperalgesia through suppression of VAL CaMKIIα‐positive neuronal activity, whereas its therapeutic effects on spontaneous and abdominal mechanical allodynia likely involve VAL CaMKIIα‐positive neuron‐independent mechanisms. These findings systematically bridge stress‐induced hyperalgesia pathophysiology, thalamic circuit modulation, and non‐pharmacological pain intervention strategies.

Mental disorders may amplify pain perception, resulting in widespread pain responses (multi‐modality and multisite manifestations) (Kroenke et al. 2010). Therefore, in the current study, we comprehensively assessed multimodal pain phenotypes—including mechanical allodynia, thermal hyperalgesia, and cold sensitivity—across multiple anatomical regions (bilateral hindpaws, forepaws, and abdominal areas) while quantifying spontaneous pain‐related behaviors. This systematic characterization of CRS‐induced nociceptive alterations validates the CRS model as a robust preclinical paradigm for investigating chronic stress‐driven pain hypersensitivity.

Neuroimaging findings suggest that aberrant functional connectivity of the anterior cingulate cortex (ACC) in anxiety disorders may amplify pain perception through disrupted integration of emotional and interoceptive signals (Yu et al. 2023). Building on this, our team employed viral tracing techniques to map whole‐brain projections from the ACC, revealing the VAL as a major downstream hub receiving dense ACC efferents (X. Ma et al. 2022; He et al. 2024). This anatomical connectivity positions VAL as a critical relay for ACC‐mediated nociceptive processing, particularly in contexts of chronic stress‐driven pain hypersensitivity. Additionally, accumulating evidence highlights the VAL as a sensorimotor hub critically engaged in nociceptive processing (Zahr et al. 2020). Functionally, VAL operates within cortico‐basal ganglia‐thalamic circuits to integrate emotional–motivational drives with pain perception (Haber and Calzavara 2009), potentially amplifying nociceptive signals in chronic stress states. Anatomically, spinothalamic pain fibers relay nociceptive inputs to VAL via brainstem nuclei (Habig et al. 2023). From VAL, these nociceptive signals are further transmitted to higher‐order cortical regions, including the primary/secondary somatosensory cortices and primary motor cortex (Craig 2002).

Thus, VAL's unique position as a convergence zone for ascending nociceptive pathways and descending cortico‐thalamic modulatory inputs underscores its dual role in the following: (1) signal relay: transmitting somatic threat signals to sensory‐motor cortices for perceptual encoding, and (2) affective integration: embedding nociception with emotional valence through interactions with stress‐related networks. This framework aligns with our findings that VAL hyperactivity exacerbates chronic stress‐driven pain hypersensitivity, suggesting its pivotal role in translating psychological distress into somatic hypersensitivity.

Following the analysis of VAL's role in pain processing, we further explore its involvement in acupuncture analgesia. Our study identified the VAL—a composite of the ventral lateral (VL) and ventral anterior (VA) nuclei—as a critical mediator in acupuncture treatment for chronic stress‐driven pain hypersensitivity. Here, we discuss potential mechanisms underlying VAL's participation in this process.

Acupuncture analgesia is proposed to modulate the sensory‐discriminative dimension of pain through the lateral pain pathway (Kato et al. 2022). Classically, this pathway includes the lateral thalamus, and primary and secondary somatosensory cortices, which collectively encode pain intensity, location, and quality (Yao et al. 2023). Anatomical studies in rodents demonstrate that VAL neurons project topographically to the primary motor cortex (M1) and are essential for sensorimotor integration (Tlamsa and Brumberg 2010). While the classical lateral pain pathway emphasizes thalamic projections to somatosensory cortices (F. Luo and Wang 2008), emerging evidence highlights the broader role of thalamic nuclei in bridging sensory and motor networks. For instance, the VL, traditionally associated with motor control, also integrates nociceptive inputs from the spinal cord (Davis et al. 2016). Notably, recent studies have revealed that the somatosensory cortex can modulate pain processing through its projections to the motor cortex, which in turn activates a descending inhibitory pathway to alleviate pain hypersensitivity (Wang et al. 2025).

These findings reveal a fundamental divergence in the mechanisms underlying the analgesic effects of EA.​​ Specifically, while EA alleviates hindpaw mechanical allodynia and thermal hyperalgesia through VAL‐dependent pathways, its ameliorative effects on abdominal mechanical allodynia ​​are mediated independently of VAL modulation. Instead​​, this effect ​​may involve compensatory pathways​​, including the following: ​​(1)​​ thalamic reorganization via normalization of hyperactivity in the ventral posterolateral nucleus (VPL) ​​(Rong et al. 2015) and paraventricular thalamic nucleus (PVT)‐dependent integration of chronic stress signals with nociceptive inputs (Dou et al. 2023), and (2) descending inhibition mediated by the periaqueductal gray (PAG)–rostral ventromedial medulla (RVM) pathway. This brainstem mechanism entails enhanced endogenous cannabinoid signaling in the PAG (N. Ma et al. 2023), which suppresses glutamatergic output to the RVM (Alam and Chen 2023). Consequently, EA ​​may recruit​​ distinct thalamic (VPL/PVT) and brainstem (PAG‐RVM) circuits to alleviate abdominal mechanical allodynia, contrasting with the VAL‐dependent mechanisms responsible for its effects on hindpaw mechanical and thermal hypersensitivity.​ ​

Collectively, VAL and its interconnected networks—spanning ascending nociceptive inputs (spinal cord), affective cortical regions (ACC), and downstream motor targets (M1)—are intricately linked to both emotion and pain processing. The convergence of these pathways within VAL provides a neuroanatomical substrate for its dual role in mediating chronic stress‐driven pain hypersensitivity, bridging pathological pain amplification (sensory dimension) and anxiety‐associated states (emotional dimension). Our findings, alongside prior evidence of M1's layer‐specific contributions to hyperalgesia (Layer 5) and negative emotion (Layer 6) (Gan et al. 2022), as well as its regulation of anxiety‐like behaviors (Z. Luo et al. 2025; Shi et al. 2023), strongly support the scientific rationale for acupuncture targeting VAL to alleviate chronic stress‐driven pain hypersensitivity. Given VAL's centrality in this integrated circuit, future research should prioritize dissecting its molecular, cellular, and circuit‐level mechanisms to refine therapeutic strategies for pain‐affective comorbidities.

An important consideration is that this study exclusively utilized male mice. This initial investigation into hyperalgesia using the CRS model focused on establishing the phenotype in males, who generally demonstrate greater baseline stability in such behavioral paradigms. However, it is well‐recognized that significant sex differences exist in stress responses and pain processing, potentially mediated by factors including estrous cycle fluctuations and hormonal influences on both nociception and stress reactivity. Therefore, the exclusive use of males represents a limitation, as the findings may not fully generalize to females. Future phases of this research program will prioritize the rigorous investigation of sex differences, carefully accounting for these critical variables (e.g., estrous cycle stage, hormonal profiles). This systematic exploration of sex‐specific mechanisms will provide more comprehensive data, ultimately enhancing the clinical translatability of the findings.

The dissociation between static and dynamic cold sensitivity in our CRS model warrants further discussion. While CRS induced robust mechanical allodynia and thermal hyperalgesia, we observed no significant change in static cold sensitivity (4°C cold plate test). This aligns with Bardin et al. (2009), who similarly reported that CRS did not alter sensitivity to sustained cold stimuli (tail immersion test at 4°C or 15°C). In contrast, neuropathic pain models (e.g., spinal nerve ligation) consistently induce static cold hyperalgesia (Ji et al. 2007), highlighting distinct pathophysiological mechanisms between stress‐induced and nerve‐injury‐induced pain hypersensitivity. Conversely, dynamic cold stimuli (e.g., acetone evaporation) are robustly enhanced by CRS (Bardin et al. 2009) and engage a central–peripheral interplay involving TRPV1‐dependent substance P release from TRPM8+ sensory neurons (F. Li et al. 2019). This mechanistic dichotomy underscores that CRS‐driven central plasticity preferentially amplifies dynamic cold pain pathways (acetone‐test responsive) but not static cold responses, which rely more heavily on peripheral TRPM8 transduction. Our findings thus emphasize the importance of stimulus modality selection when evaluating cold hypersensitivity in stress‐related pain models.

While deep brain stimulation targeting the ventral anterior/ventrolateral thalamus (VAL) is primarily implemented clinically for movement disorders such as Parkinson's disease, serendipitous observations during routine postoperative assessments revealed its ancillary benefit in alleviating anxiety symptoms (Huys et al. 2016). This unexpected therapeutic effect highlights VAL's broader neuromodulatory potential beyond motor circuitry. Nevertheless, clinical applications of VAL‐DBS specifically for anxiety or pain conditions remain exceptionally limited in current practice. This scarcity stems predominantly from two critical knowledge gaps: (1) a paucity of foundational studies elucidating VAL's role in affective and nociceptive processing, and (2) incompletely defined mechanisms underlying its non‐motor effects. Our investigation represents an initial exploration of VAL circuitry in stress‐pain comorbidities. By establishing mechanistic links between VAL modulation and stress‐induced pain hypersensitivity in preclinical models, these findings address the aforementioned research gaps. We anticipate this work will catalyze future translational studies, potentially paving the way for targeted clinical trials of VAL neuromodulation in pain conditions with stress comorbidity.

## 5 Conclusions

This study identifies VAL CaMKIIα‐positive neurons as critical mediators of chronic stress‐induced pain hypersensitivity. EA alleviates chronic stress‐driven mechanical and thermal hyperalgesia in the hindpaws through suppression of these neurons, while relief of spontaneous pain and abdominal mechanical allodynia involves distinct neural mechanisms. These findings delineate the role of the thalamic VAL in emotion–pain comorbidity and validate non‐pharmacological neuromodulation as a targeted therapeutic strategy for chronic stress‐associated pain disorders.

## Author Contributions

**Jie Chen**: methodology, formal analysis. **Qinling Li**: methodology, data curation. **Wei Yu**: methodology, validation. **Min He**: methodology, validation. **Zui Shen**: conceptualization, writing–review and editing, funding acquisition. **Peipei Feng**: writing–original draft, funding acquisition.

## Ethics Statement

Ethical approval was granted by the Ethics Committee of Zhejiang Chinese Medical University (IACUC‐20221219‐08).

## Conflicts of Interest

The authors declare no conflicts of interest.

## Peer Review

The peer review history for this article is available at https://publons.com/publon/10.1002/brb3.70855

## Supporting information




**Supplementary Figures**: brb370855‐sup‐0001‐FigureS1‐S3.docx

## Data Availability

The data that support the findings of this study are available from the corresponding author (Zui Shen) upon reasonable request.
