# Supplementary material for: Ventral Anterior–Lateral Complex of the Thalamus Mediates Chronic Stress‐Induced Pain Hypersensitivity and Underlies Electroacupuncture Analgesia
Source: Brain Behav. 2025 Sep 9;15(9):e70855. doi: 10.1002/brb3.70855 (PMC12417967; doi:10.1002/brb3.70855)
Supplement: Supplementary file 1 — Supplementary Figures: brb370855‐sup‐0001‐FigureS1‐S3.docx [file BRB3-15-e70855-s001.docx]

| **Supplementary Figures：** |
| --- |
| 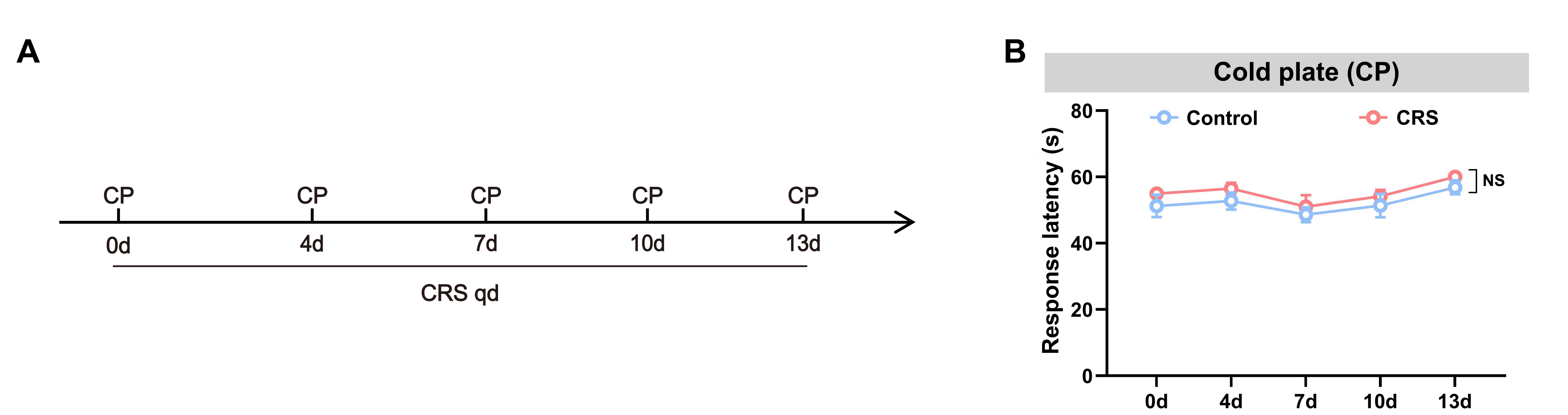 |
| **Supplementary Figure 1.** Chronic restraint stress does not affect cold hyperalgesia in the cold plate test. (A) Schematic of the experimental design. (B) Statistical results of cold plate latency. In (B): Two-way repeated-measures ANOVA. NS: not significant. |

| 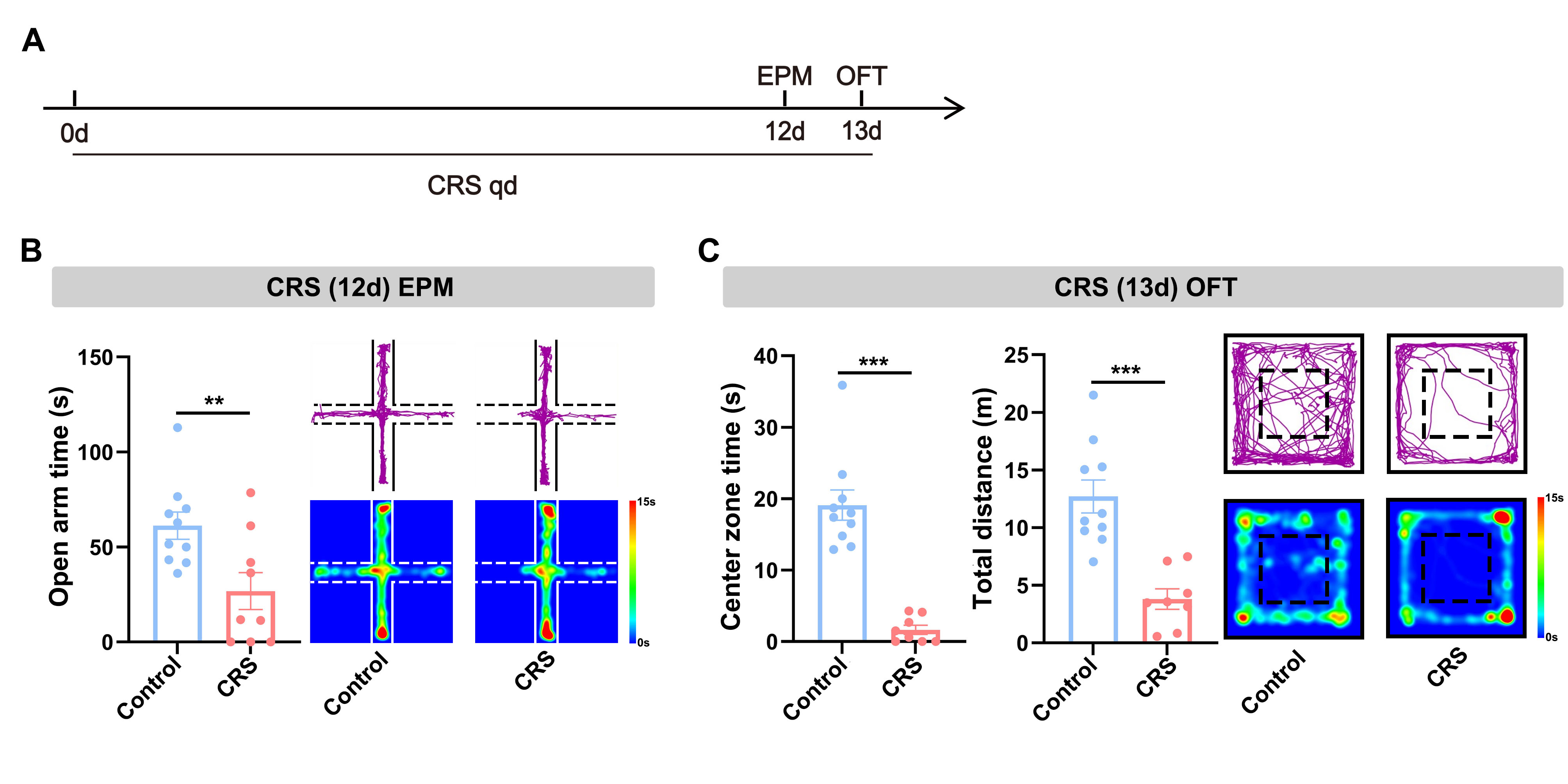 |
| --- |
| **Supplementary Figure 2.** Chronic restraint stress induced anxiety-like behaviors. (A) Schematic of the experimental design. (B) Statistical results of time spent in the open arms in the EPM test. (C) Statistical results of time spent in the central area and total distance traveled in the OFT. In (B) and (C): Independent samples t-test, ***P* < 0.01, ****P* < 0.001. EPM elevated plus-maze, OFT open field test. |

| 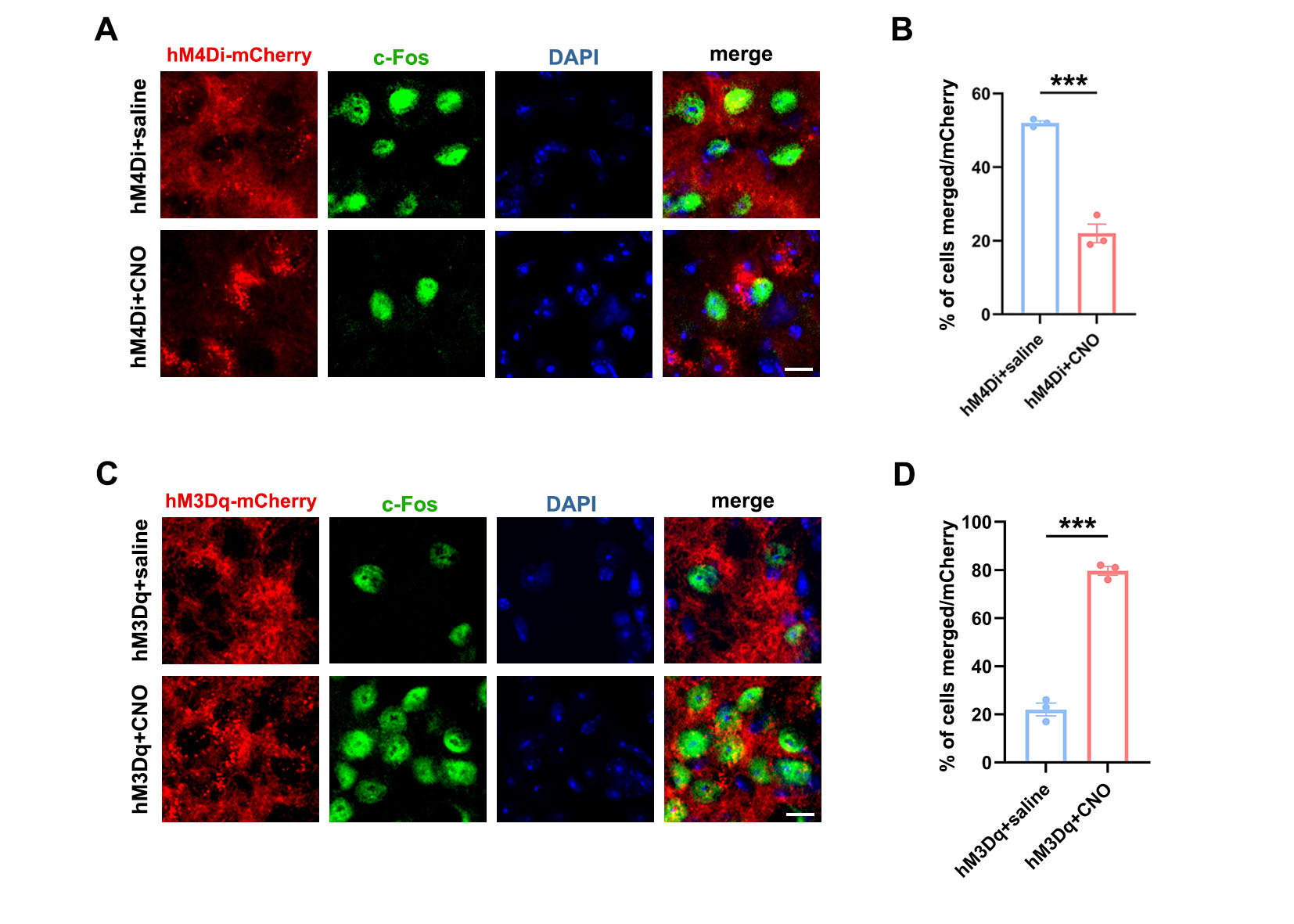 |
| --- |
| **Supplementary Figure 3.** Viral efficacy in neuronal activity modulation. (A) Representative immunofluorescence images showing CNO-induced suppression of neuronal activity via hM4Di-mCherry expression in VAL CaMKIIα-positive neurons. Co-staining for c-Fos (activity marker), hM4Di-mCherry (red), and DAPI (blue). Scale bar: 20 µm. (B) Percentage of labeled mCherry+ neurons expressing c-Fos in hM4Di +saline group and hM4Di +CNO group. (C) Representative immunofluorescence images showing CNO-induced activation of neuronal activity via hM3Dq-mCherry expression in VAL CaMKIIα-positive neurons. Co-staining for c-Fos (green), hM3Dq-mCherry (red), and DAPI (blue). Scale bar: 20 µm. (D) Percentage of labeled mCherry+ neurons expressing c-Fos in hM3Dq +saline group and hM3Dq +CNO group. In (B) and (D): Independent samples t-test, ****P* < 0.001. |
